# Assessing equity in the distribution of high-technology medical equipment in Guangxi: evidence from an ethnic minority region in Southern China

**DOI:** 10.1186/s12939-017-0568-0

**Published:** 2017-05-16

**Authors:** Jian Sun, Hai Gu, Qiulin Wen, Hongye Luo

**Affiliations:** 1 School of Humanities and Social Science, Guangxi Medical University, 22 Shuang Yong Road, Qing Xiu District, Nanning, 530021 Guangxi Zhuang Autonomous Region China; 2 Research Center of Health Policy and Management, Nanjing University, 163 Xian Lin Avenue, Qi Xia District, Nanjing, 210023 Jiangsu Province China; 3 School of Pharmacy, Guangxi Medical University, 22 Shuang Yong Road, Qing Xiu District, Nanning, 530021 Guangxi Zhuang Autonomous Region China; 4 School of Information and Management, Guangxi Medical University, 22 Shuang Yong Road, Qing Xiu District, Nanning, 530021 Guangxi Zhuang Autonomous Region China

**Keywords:** High-technology medical equipment, Equity, Distribution, Concentration index, Guangxi Zhuang Autonomous Region

## Abstract

**Background:**

High-technology medical equipment (HTME) are important health resources. However, there is unequal distribution of these equipment in favor of metropolis and well equipped health facilities. This study sought to examine the equity gaps in the distribution of HTME in Guangxi. The results of this study could shed light on the future HTME allocation in Guangxi Zhuang Autonomous Region.

**Methods:**

Data related to HTME was sourced from a general investigation of all the hospitals of Guangxi. Concentration index was used to assess the equity status of HTME in Guangxi.

**Results:**

Over all, the total amount of HTME in Guangxi had been increasing from 2011 to 2015, and the per million population HTME of five kinds were all increased at the same time. Meanwhile, the concentration indices ranged between 0.1020 and 0.4617. The five medical equipment were all concentrated among the rich.

**Conclusions:**

The possession of SPECT per million population in Guangxi is lower than the national average level while it is superior to the national average level for CT, MRI, DSA and LA. The equity status in the distribution of the five medical equipment has deteriorated since 2011. In 2015, the equity status of CT was the best, while the equity status of MRI was the worst. Meanwhile, 45.1% of HTME were concentrated in Nanning, Guilin, and Liuzhou.

## Background

According to relevant documents released by the China National Health and Family Planning Commission, the term “high-technology medical equipment” (HTME) refers to high-technology, large-scale, precise and valuable instruments Many HTME are used in a health care setting for diagnosis. Undoubtedly, HTME are important health resources, playing a prominent role in enhancing the health care quality and the accuracy of diagnoses [1–3].

Assessing equity in the distribution of HTME in Guangxi is of utmost significance for several reasons. On the one hand, there is huge gap in socio-economic development in Guangxi. Three major cities, namely, Nanning, Guilin, and Liuzhou, have more health resources, the competition among hospitals may worsen the allocation equity of HTME. On the other hand, availability of HTME plays a vital role in improving health system’s performance. However, there is unequal distribution of these equipment in favor of metropolis and well equipped health facilities, which affects the availability and the equity of HTME significantly. This study sought to examine the equity gaps in the distribution of HTME in Guangxi using concentration index. The results of this study could shed light on the future HTME allocation in Guangxi.

Though published research has reported on the inequity of health care resources allocation [4–8], only a small number of researches have focused on the equity status of HTME. For example, two studies show that CT and MRI in China based on population distribution is relatively fair while it’ s less fair based on geographical distribution [9, 10]. According to a study on the equity status of HTME in Guangxi, the Gini coefficients of CT, MRI, DSA, LA, and SPECT were smaller than 0.40, which indicated a relatively equitable allocation [11]. A study on the equity status of HTME in a province showed that the Gini coefficients of LA, DSA, SPECT were higher than 0.40, which indicated that the equity of these three equipment was in alerting status [12]. A study found that the equity status of the new models of CT, MRI, and PET got improve in Japan, while the old models got worse [13].

Guangxi Zhuang Autonomous Region is one of the five ethnic minority regions in China, and it is located in southwestern China and borders Guangdong Province, Hunan Province, Guizhou Province and Yunnan Province. Guangxi includes 14 prefecture-level cities and 8 county-level jurisdictions. Aside from the majority Han population, Guangxi has more recognized ethnic minority groups than any other region in China, including Zhuang, Yao, Miao and Dong, which accounts for 37% of its population [14, 15].

## Methods

### Data sources

Demographic, economic and social development data was obtained from the Guangxi Statistical Yearbook from 2011 to 2015 [16–20]. Data related to HTME was sourced from a general investigation in each hospital of Guangxi from 2011 to 2015.

### Investigation method and statistical analysis

We arranged for fully trained interviewers to go to each hospital of Guangxi to conduct a questionnaire survey. Through the questionnaire survey, we collected the numbers of CT, MRI, DSA, LA, and SPECT of each city in Guangxi from 2011 to 2015. Then, the interviewers verified the relevant information on site to ensure the authenticity of the survey data, and entered the data into EpiData 3.0. We calculated statistical indicators such as constituent ratio, growth rate, concentration index and drew figures using Microsoft Excel 2013.

### Concentration index

Concentration index was used to assess the degree of equity of HTME. The World bank recommends using a concentration index to assess the degree of equity of health services in different economic and social status groups [21]. We selected CT, MRI, DSA, LA and SPECT as objects of investigation [22]. The following formula was employed to calculate the concentration index:

$$ \mathrm{S}=\frac{1}{2}{\displaystyle \sum_{\mathrm{i}=0}^{\mathrm{n}-1}\left(\mathrm{Yi}+\mathrm{Yi}+1\right)\left(\mathrm{Xi}+1-\mathrm{Xi}\right)} $$

$$ \mathrm{C}\mathrm{I}=2\times \left(0.5-\mathrm{S}\right), $$

where Y_0_ is equal to zero and X_0_ is equal to zero; Y_i_ is the cumulative proportion of HTME, X_i_ is the cumulative proportion of population, and i is the fractional rank according to per capita GDP beginning with the lowest; CI represents the concentration index [23].

The concentration index ranges between −1 (pro-poor) and +1 (pro-rich); the greater the absolute value of concentration index, the greater the degree of inequities; a value of zero indicates absolute equity; a negative value indicates a concentration of HTME on the poorer populations; a positive value indicates a concentration of HTME on the richer populations [24–27].

## Results

### Basic information of HTME allocation of Guangxi from 2011 to 2015

**Fig. 1:**
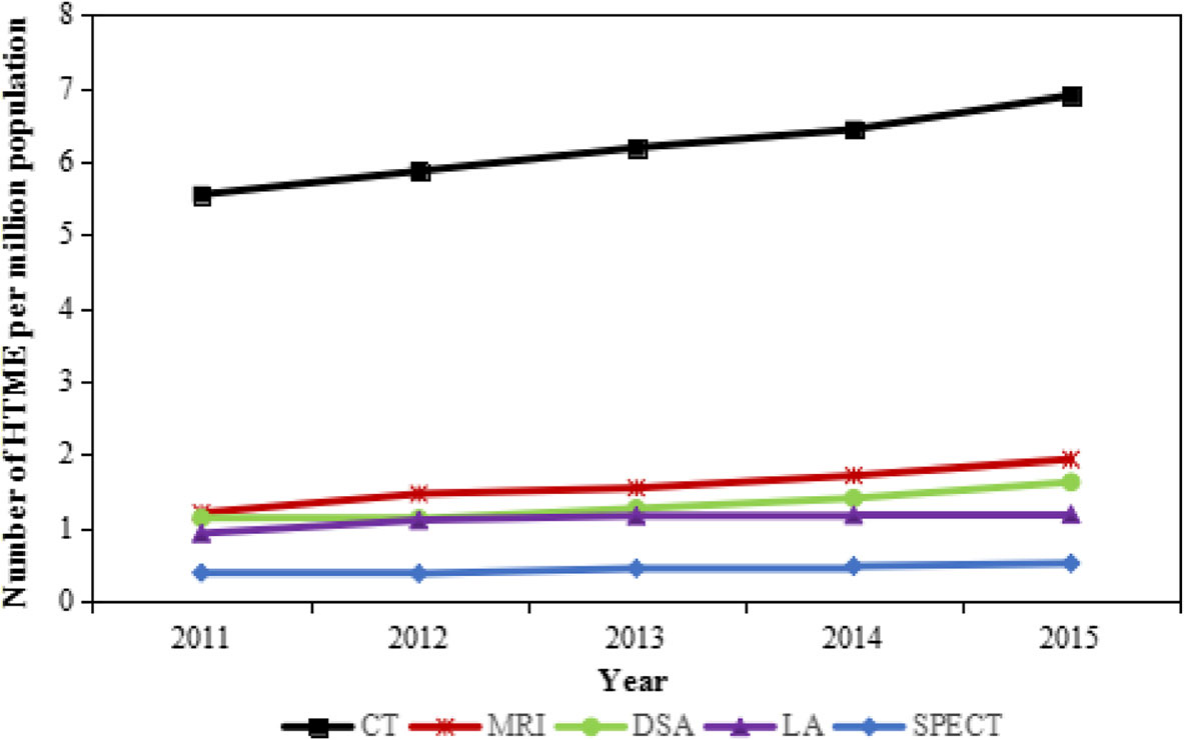
Number of HTME per million population from 2011 to 2015

Basic information of HTME allocation from 2011 to 2015 were shown in Table 1 and Fig. 1. Over all, HTME in Guangxi had been increasing from 2011 to 2015, and the per million population HTME of five kinds were all increased at the same time. Per million population number of CT, MRI, DSA, LA, and SPECT rose 24.32, 60.33, 42.98, 27.96, and 33.33% respectively. Meanwhile, the basic figure of population had been grown from 46.45 to 47.96 million, and per capita GDP increased from 25,233.30 RMB to 35,035.70 RMB.

**Table 1:** Number of HTME per million population from 2011 to 2015

Year	Population (10,000 persons)	Per capita GDP (RMB)	Number of HTME per million population				
			CT	MRI	DSA	LA	SPECT
2011	4,645.00	25,233.30	5.55	1.21	1.14	0.93	0.39
2012	4,682.00	27,840.88	5.87	1.47	1.13	1.11	0.38
2013	4,719.00	30,620.68	6.19	1.55	1.27	1.17	0.45
2014	4,754.00	32,967.80	6.44	1.72	1.41	1.18	0.48
2015	4,796.00	35,035.70	6.90	1.94	1.63	1.19	0.52

### Regional distribution of HTME in 2015

**Fig. 2:**
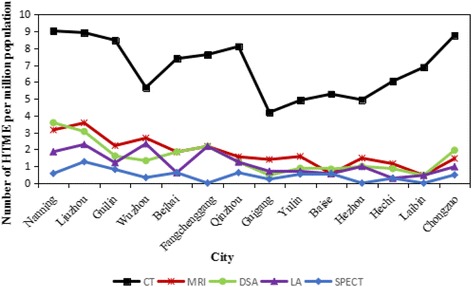
Number of HTME per million population in Guangxi in the year of 2015

In order to make a deeper understanding of the HTME distribution in Guangxi, we made a calculation of the number of HTME per million population in 2015. The specific values were show in Table 2 and Fig. 2. In 2015, Guangxi had 584 HTME, including 331 CT, 93 MRI, 78 DSA, 57 LA, and 25 SPECT, and the number per million population was 6.90, 1.94, 1.63, 1.19, and 0.52, respectively. The average annual growth rates for these equipment were 6.4, 13.5, 10.1, 7.3, and 8.6% from 2011 to 2015, respectively. Among the study sites, Liuzhou had the highest number of HTME per million population in 2015 (19.31) while Guigang had the lowest level (7.04).

**Table 2:** Number of HTME per million population in Guangxi in the year of 2015

City	Population (10,000 persons)	Per capita GDP (RMB)	Number of HTME per million population				
			CT	MRI	DSA	LA	SPECT
Nanning	698.61	49,066	9.02	3.15	3.58	1.86	0.57
Liuzhou	392.27	58,869	8.92	3.57	3.06	2.29	1.27
Guilin	496.16	39,327	8.47	2.22	1.61	1.21	0.81
Wuzhou	299.94	36,106	5.67	2.67	1.33	2.33	0.33
Beihai	162.57	55,239	7.38	1.85	1.85	0.62	0.62
Fangchenggang	91.84	67,971	7.62	2.18	2.18	2.18	0.00
Qinzhou	320.93	29,560	8.10	1.56	1.25	1.25	0.62
Guigang	429.37	20,240	4.19	1.40	0.47	0.70	0.23
Yulin	570.72	25,440	4.91	1.58	0.88	0.70	0.53
Baise	359.67	27,365	5.28	0.56	0.83	0.56	0.56
Hezhou	202.59	23,178	4.94	1.48	0.99	0.99	0.00
Hechi	347.68	17,841	6.04	1.15	0.86	0.29	0.29
Laibin	218.20	25,677	6.87	0.46	0.46	0.46	0.00
Chongzuo	205.45	33,355	8.76	1.46	1.95	0.97	0.49

### Analysis of concentration index

Table 3 compares concentration indices for the five medical equipment from 2011 to 2015. The concentration indices ranged between 0.1020 and 0.4617: 0.1020–0.1197 for the number of CT, 0.2064–0.4617 for the number of MRI, 0.2971–0.3084 for the number of DSA, 0.2436–0.2514 for the number of LA, 0.1988–0.2201 for the number of SPECT. The concentration indices of the five medical equipment were positive, which indicates a disproportionate concentration of the equipment among the rich.

**Table 3:** Concentration index of HTME in Guangxi from 2011 to 2015

Year	CT	MRI	DSA	LA	SPECT
2011	0.1020	0.2064	0.2971	0.2436	0.1988
2012	0.1037	0.2530	0.2831	0.2470	0.1990
2013	0.1102	0.2217	0.3021	0.2501	0.2100
2014	0.1135	0.3015	0.3050	0.2510	0.2180
2015	0.1197	0.4617	0.3084	0.2514	0.2201

**Fig. 3:**
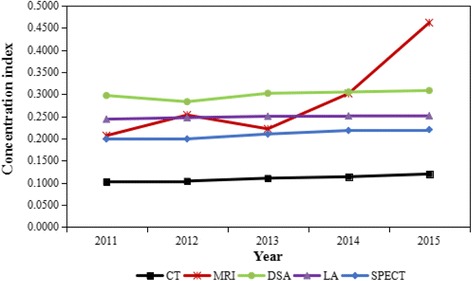
Concentration index of HTME in Guangxi from 2011 to 2015

Figure 3 shows the time trend of concentration index for the five medical equipment from 2011 to 2015. From 2011 to 2015, the concentration index of the five medical equipment showed an overall upward trend. The concentration index of MRI experienced the largest increase (123.69%), more than 38 times the increase for LA (3.20%). At the same time, the concentration index of CT was significantly lower than other equipment, indicating that the equity status was the best. On the contrary, the concentration index of MRI was higher than other equipment in the year of 2015, which indicates that the equity status was the worst.

## Discussion

From the absolute number point of view, the quantity of CT, MRI, LA in Guangxi is inferior to Hunan Province (372 CT, 103 MRI, 66 DSA, 63 LA, and 19 SPECT) in central China while it exceeds that of Hunan for DSA and SPECT. Furthermore, the total number of HTME in Guangxi exceeds the number in Xinjiang Uyghur Autonomous Region (218 HTME) in Western China. The possession of SPECT per million population in Guangxi is lower than the national average level while it is superior to the national average level for CT, DSA, MRI and LA. The possession of CT and MRI per million population in Guangxi is inferior to that of Shanghai (7.60 CT per million population, 3.23 MRI per million population), a developed independent municipality in Eastern China.

Overall, the concentration indices for CT, MRI, DSA, LA, and SPECT increased with growth rates of 17.35, 123.69, 3.80, 3.20, and 10.71% respectively from 2011 to 2015, which indicates that the equity status of the five medical equipment worsened. This may be partially due to the fact that those equipment are more concentrated in Nanning, Guilin, and Liuzhou, where rates increased from 43.7% in 2011 to 45.1% in 2015.

In 2015, the ranking of the equity status from the best to the worst was CT, SPECT, LA, DSA, and MRI. The distribution of CT was the fairest, which was consistent with the findings of Zhu [11] and He [9, 12]. CT was introduced into China early and has become commonly used in the examination of many diseases in hospitals in both underdeveloped and developed areas, thus the distribution of CT was fairest [10]. Conversely, the distribution of MRI was the most unfair. For the differences in economics, hospitals in economically underdeveloped areas are not able to afford MRI, and since 50.5% of all MRI are concentrated in three major cities, namely Nanning, Guilin, and Liuzhou, the distribution of MRI was the least fair.

Based on our analysis, we put forward the following advice to improve the overall equity of HTME allocation. First, the Department of Health should formulate and implement regional health planning of HTME, and implement severe penalties for any hospital that fails to follow its guidelines [28]. In addition, the Department of Health should increase financial support to the areas that have a relatively lower number of HTME per million population to improve the accessibility and equity of health services. In the meanwhile, it is necessary for the Department of Health to control the number of HTME in some cities that have a relatively higher number of HTME per million population, and to pay more attention to the equity status of MRI. Second, medical institutions at various levels should abide by the regulations released by the Department of Health. According to the actual situation of the medical institutions and the needs of local residents, medical institutions at various levels should allocate HTME rationally. Third, hospitals should set up regional image diagnosis and treatment centers and explore mechanisms for sharing HTME in order to improve their utilization and efficiency [29, 30].

## Conclusion

Inequity in the distribution of HTME still exists in Guangxi. Overall, the equity status in the distribution of the five medical equipment has deteriorated since 2011. To reduce the inequity of HTME in Guangxi, stakeholders, including policymakers, hospitals, and patients, should strive to cooperate jointly in order to ameliorate the situation.

## Abbreviations

CT: Computed tomography
DSA: Digital subtraction angiography
HTME: High-technology medical equipment
LA: Linear accelerators
MRI: Magnetic resonance imaging
SPECT: Single photon emission computed tomography

## References

[1] Sun J, Zhu P (2016). Configuration and utilization of class B large medical equipment in Guangxi. Mod Prev Med.

[2] Zhu P, Wang Q (2010). Research progress of configuration and management of large medical equipment. Chin Health Econ.

[3] Chen Y, Geng J, Wu B (2016). Evaluation on high-tech medical equipment regulation policy and suggestions. Chin Hosp Manage.

[4] Zhang T, Xu Y, Ren J (2017). Inequality in the distribution of health resources and health services in China hospitals versus primary care institutions. Int J Equity Health.

[5] Jin J, Wang J, Ma X (2015). Equality of medical health resource allocation in China based on the Gini coefficient method. Iran J Public Health.

[6] Horev T, Pesis-Katz I, Mukamel DB (2004). Trends in geographic disparities in allocation of health care resources in the US. Health Policy.

[7] Zhang X, Zhao L, Cui Z (2015). Study on equity and efficiency of health resources and services based on key indicators in China. PLoS One.

[8] Omranikhoo H, Lotfi F, Safari H (2013). Equity in distribution of health care resources: assessment of need and access, using three practical indicators. Iran J Public Health.

[9] He D, Yu H, Chen Y (2013). Equity in the distribution of CT and MRI in China: a panel analysis. Int J Equity Health.

[10] Wu B, Chen Y, Geng J (2016). An equity analysis of CT and MRI in Eastern, Middle and Western China. Chin Hospital Manage.

[11] Zhu P (2012). Equity evaluation on allocation of Class B medical equipment in Guangxi Zhuang Autonomous Region. Med Soc.

[12] He D, Liu J, Chen Y (2012). Analyzing the equity of allocating large medical equipment of class B. Chin Health Serv Manage.

[13] Matsumoto M, Koike S, Kashima S (2015). Geographic distribution of CT, MRI and PET devices in Japan: a longitudinal analysis based on national census data. PLoS One.

[14] Huang L, Yang D, Yao L (2013). Guangxi’s rural health insurance scheme: evidence from an ethnic minority region in China. Rural Remote Health.

[15] Wang X, Luo H, Qin X (2016). Evaluation of performance and impacts of maternal and child health hospital services using data envelopment analysis in Guangxi Zhuang Autonomous Region, China: a comparison study among poverty and non-poverty county level hospitals. Int J Equity Health.

[16] Guangxi Bureau of Statistics. Guangxi Statistical Yearbook 2012. Beijing: China Statistics Press; 2012.

[17] Guangxi Bureau of Statistics. Guangxi Statistical Yearbook 2013. Beijing: China Statistics Press; 2013.

[18] Guangxi Bureau of Statistics. Guangxi Statistical Yearbook 2014. Beijing: China Statistics Press; 2014.

[19] Guangxi Bureau of Statistics. Guangxi Statistical Yearbook 2015. Beijing: China Statistics Press; 2015.

[20] Guangxi Bureau of Statistics. Guangxi Statistical Yearbook 2016. Beijing: China Statistics Press; 2016.

[21] Shen C, Tao X, Dong W (2015). Analysis on health resources allocation equity with concentration index method in Xi’an city. Chin J Health Policy.

[22] Zhang X, Zhao L, Qing X (2014). Equity analysis of health resources allocation with the concentration index method for provinces in China. Chin J Hosp Adm.

[23] Liang S, Feng Q, Wang Y (2015). Studying on the equity of health resources allocation in the minority nationality areas sampled with Guangxi. Chin Health Serv Manage.

[24] Zhou Z, Su Y, Gao J (2013). Assessing equity of healthcare utilization in rural China: results from nationally representative surveys from 1993 to 2008. Int J Equity Health.

[25] Chen M, Palmer AJ, Lei S (2016). Assessing equity in benefit distribution of government health subsidy in 2012 across East China: benefit incidence analysis. Int J Equity Health.

[26] Yuan S, Rehnberg C, Sun X (2014). Income related inequalities in new cooperative medical scheme: a five-year empirical study of Junan county in China. Int J Equity Health.

[27] Huang Y, Huo H, Wu W (2016). Equity analysis on food and drug inspection resources allocation in Guangxi. Chin Health Resour.

[28] Wang Q, Peng Y, Su J (2011). Allocation and analysis for large medical equipment in Guangxi. Chin Health Resour.

[29] Zhao Z (2015). Assessment and forecasting of the12th five-year plan of large medical equipments in Hangzhou. Chin Health Resour.

[30] Guan B, Xu H (2015). Optimal configuration and management of large medical equipment in hospital. Hosp Adm J Chin People’s Liberation A!rmy.

